# Twists and turns: 40 years of investigating how and why bacteria swim

**DOI:** 10.1099/mic.0.001432

**Published:** 2024-02-16

**Authors:** Judith P. Armitage

**Affiliations:** ^1^​ Department of Biochemistry, University of Oxford, OX1 3QU, UK

**Keywords:** chemotaxis, memoir, motility, Rhodobacter, swimming

## Abstract

Fifty years of research has transformed our understanding of bacterial movement from one of description, based on a limited number of electron micrographs and some low-magnification studies of cells moving towards or away from chemical effectors, to probably the best understood behavioural system in biology. We have a molecular understanding of how bacteria sense and respond to changes in their environment and detailed structural insights into the workings of one of the most complex motor structures we know of. Thanks to advances in genomics we also understand how, through evolution, different species have tuned and adapted a core shared system to optimize behaviour in their specific environment. In this review, I will highlight some of the unexpected findings we made during my over 40-year career, how those findings changed some of our understanding of bacterial behaviour and biochemistry and some of the battles to have those observations accepted.

## Introduction

Forty years ago we knew that most bacteria swim at least some of the time, periodically changing swimming direction to bias their overall movement toward a more favourable environment. It had also just become clear that swimming is dependent on the transmembrane proton gradient rotating motors embedded in the cytoplasmic membrane attached to extracellular semi-rigid helical flagella filaments, making bacterial swimming completely different from that seen in eukaryotic microbes. Because bacteria are so small it was clear they could not sense a spatial gradient but rely on temporal sensing of changes in effector concentration to control their direction changing frequency. In *Escherichia coli* this was found to depend on sensing a limited number of attractants through binding to transmembrane receptors that both signal to the motor via a diffusible protein and then adapt to that signal [1]. Changing the size and frequency of stimuli revealed the chemosensory system has both phenomenal sensitivity and a memory that allows bacteria to respond to a very small percentage change in background concentration over orders of magnitude [2].

It was fortunate that most early research used *Escherichia coli* as it turns out to have both a relatively basic chemosensory and motor system. While these basic systems are conserved across all swimming bacterial groups, pointing to an early evolutionary origin, evolution has built on these systems so that now most bacterial species responding to multiple stimuli, often through multiple pathways to balance a tactic response suited to their natural environment [3]. The core structure of the motor is also common across all bacteria, but many species have added complexity allowing, for example, increased torque in viscous environments or the use of sodium rather than protons as the driving force [4, 5]. The rotational mechanism appears conserved, rotating a central core against a ring of membrane-associated proteins.

Among the many surprises the motility system has sprung over the years is the dynamic nature of the motor proteins. We now know that many large protein complexes show dynamic exchange of some or all of their proteins, and the composition can be altered in response to changes in the environment requiring changes in output, but the motor was the first structure where this remodelling was revealed. In the following few pages, I will describe some of the, often serendipitous, discoveries we made over the years and hopefully show that following up unexpected, but repeatable results, can produce insights that can overturn current thinking.

## ‘Seeing’ a 45 nm nanomachine working

### Limitations to swimming and sensing

In 1676 Van Leuwenhoek, using his single lens microscope, was the first person we know of to describe bacteria in one of the numerous letters he wrote to the Royal Society. He identified these tiny objects as living creatures, animalcules, because they moved very rapidly and with purpose. Non-living or non-motile particles of the same size just jiggle around as they are buffeted by solvent molecules, Brownian motion. Brownian motion gives an insight into the unusual world in which bacteria must move and sense. There is no inertia in the bacterial world, just viscosity. The relationship between inertia and viscosity on an organism’s movement is described as the Reynolds number. A swimming human has a Reynolds number of about 10^6^, a tanker around 10^9^, but for a swimming bacterium it is about 10^−6^ . Bacteria do not displace the medium around them as they swim and when they stop there is no continued forward momentum, they just stop. However, because of the molecules around them are in constant motion they are being constantly bombarded and pushed off course by this Brownian motion. When you look at motile bacteria down a microscope you see a random three-dimensional pattern of swimming and direction changing. This is because bacteria, in general, are too small to sense spatial gradients and therefore must compare now with a few seconds ago, changing direction regularly to account for Brownian motion and gradient changes [6, 7]. This three-dimensional swimming pattern, with random changes in swimming direction made tracking swimming extremely difficult as microscopes have limited depth of focus and 40 years ago computers were unable to track multiple moving objects.

### The flagellar structure

**Fig. 1.**
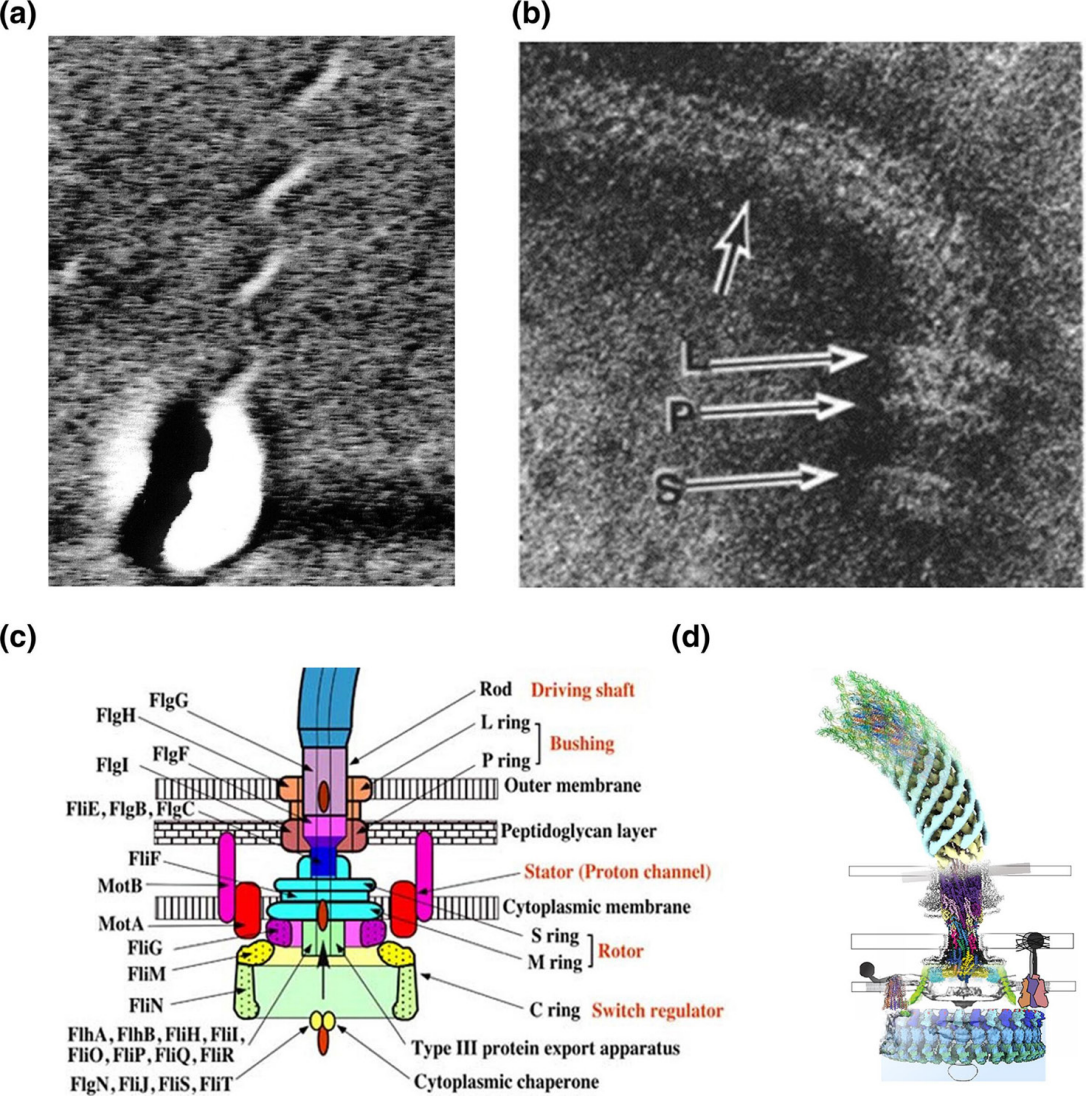
Development of our understanding of the structure of the flagellum and its motor. (a) An electron micrograph of *Rhodobacter sphaeroides* with a single filament; (b) a negative stain of the *E. coli* motor and filament from a lysed cell showing the L and P rings in the outer membrane and S shows what is now the C, or MS, motor ring in the cytoplasmic membrane (from [58] with permission); (c) the components of the motor as understood at the beginning of the twentieth century. Left side gives protein names, unified across species, right side the structural components formed from the proteins and in red their function [59]; (d) the 2020 cryoelectron tomography structural model of the flagellar motor showing on stator attached to the wall and another disengaged [12].

The flagellum is the product of a regulon, or coordinated operons, of around 50 genes sequentially expressing about 25 proteins that assemble sequentially to form an ordered nanomachine across the bacterial membrane and cell wall, from the cytoplasm to several micrometres into the outside environment [8, 9]. Assembling such a complex structure from the cytoplasm all the way to extracellular requires complex choreography as expressing one component before the right export and scaffolding structures are in place could lead to cell damage or death. While the core inner-membrane proteins use the classical sec transport system and the outer-membrane ring the OMP export pathway, the extracellular components are exported through an ATPase-dependent type 3 secretion system (T3SS) [10]. This export apparatus is responsible for sequentially exporting the rod, which attaches the rotor to the extracellular hook. Once a hook of precisely defined length has been built, the filament proteins are expressed and exported, travelling unfolded up a central channel in the hook and filament to polymerize at the distal end, helped by hook and filament capping proteins (Fig. 1). Thousands of FliC flagellin proteins polymerize to form a semi-rigid filament with a species-specific helical wavelength and handedness [11].

Extensive recent structural studies have culminating in a number of cryoEM high-resolution structures revealing the detailed structure of the motor. The key structural ring, FliF shares a 34-fold symmetry with FliG, the core rotor protein. FliG interacts on its cytoplasmic face with FliM and FliN that drive motor switching in response to binding by the chemosensory signalling protein CheY released by environmental signals. The export apparatus sits at the base of these rings. The FliG rotor ring is surrounded by a ring of transmembrane Mot stator proteins, each with a ring of 5 MotAs surrounding 2 MotBs. These stators are the driving units of the motor using the transmembrane ion gradient to drive rotation, and will be discussed in more detail below (Fig. 1) [12].

Rotation of majority of flagella investigated have been shown to be driven by the transmembrane electro-chemical proton gradient, formed across the membrane by respiration, photosynthesis or reverse ATPase activity during fermentation [13]. However bacterial species living in alkaline or high sodium environments often use the transmembrane sodium gradient, with some species using either. Rotation can be as high as 300 revolutions per second for a proton driven motor, moving a bacterium through its environment at tens of micrometres per second, some species moving as fast as 100 µm s^–1^. As bacteria experience high levels of viscosity when swimming the architecture of the motor is optimized in different species to specific environments to optimize the torque output [4].

## Modifying the motor in response to the environment

Back in the late 1960s it was realized that the motor was rotary, unlike the whiplash of eukaryotic microbes, because if a slide is coated with anti-flagellin antibody bacteria will attach to the slide via a flagellar filament and the cell bodies will rotate smoothly around one spot, switching direction of rotation periodically. This can only happen if there is a rotary motor at the base of the flagellum [14]. These tethered cells are the basis of many experiments studying both flagella function and chemosensory control of rotation. One early investigation of the motor structure used mutants with the stator (*mot*) genes deleted. Mot mutants still produced flagella, but are paralysed. However, expression of the *mot* genes from a plasmid resulted in a step-wise increase in rotation of the tethered cell body. This showed not only that the Mot proteins are the stator proteins essential for motor rotation but that individual stators could incorporate and drive some rotation and, as it took 8–13 steps to reach full rotation speed, there are probably 8–13 stators around the rotor [15].

Rotating a tethered cell body requires high torque as the large cell body is pushed against the viscous environment, while swimming in liquid requires much lower torque. We and others showed later that the number of stators required to rotate a filament depends on the viscosity of the environment. Extensive biochemical and genetic evidence suggested that MotB of the stator has two conformations, a folded and an extended form [16]. Extension of the periplasmic domain of MotB allows it to bind the peptidoglycan, opening a proton channel. When protons move down the transmembrane gradient they bind to Asp32 on MotB changed the conformation of MotA, possibly causing rotation and a change in electrostatic interaction with the rotor protein FliG, and driving the rotor rotation, with several hundred protons per second flowing through the ring of Mot complexes for maximum rotational speed.

**Fig. 2.**
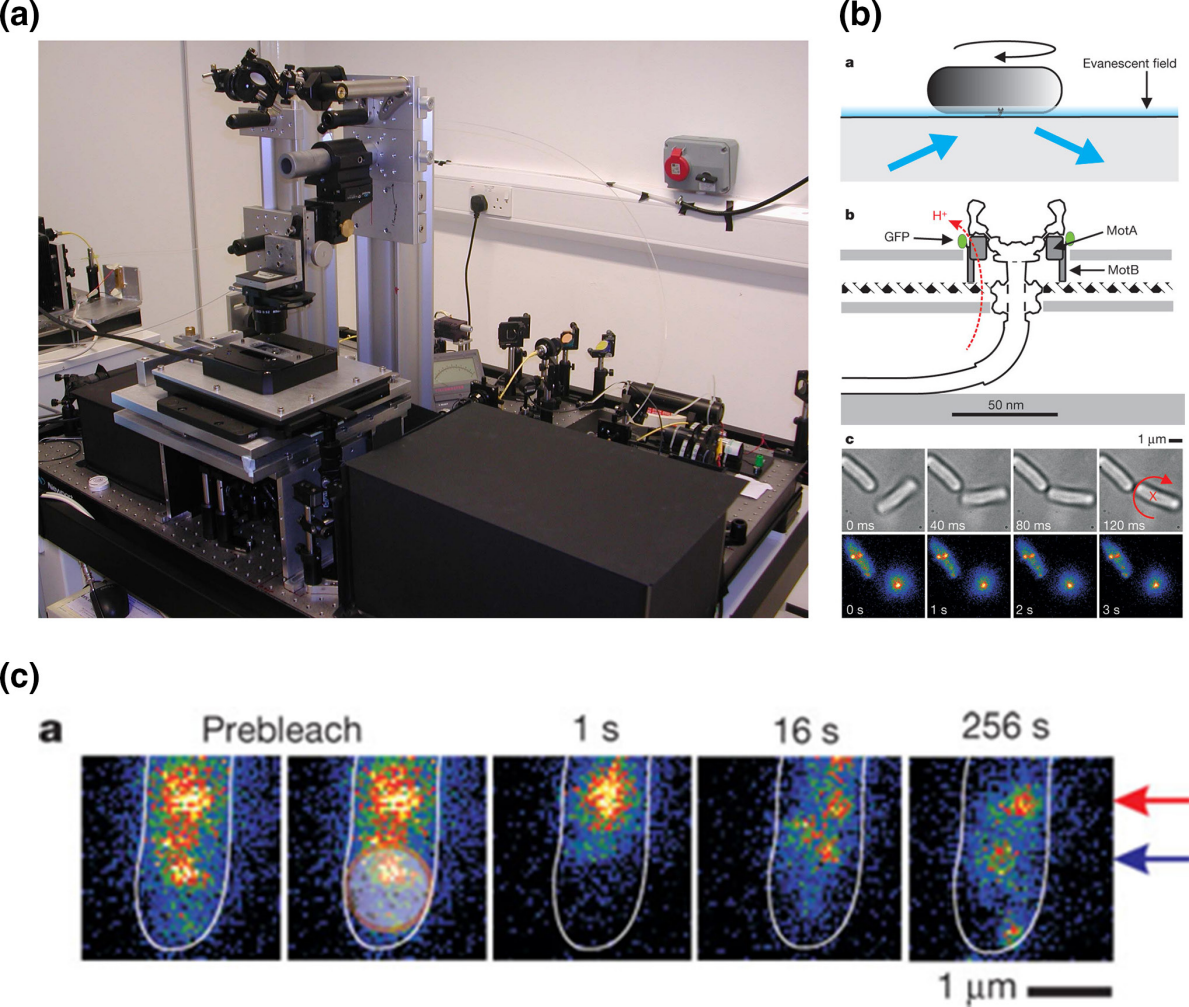
. TIRF experiment showing stator exchange. (a) Home-built TIRF microscope; (b)a *E. coli* tethered by its flagellum showing evanescent field, (b)b GFP-MotB fusions pulled within the field, (b)c two cell with GFP-labelled stators, one rotating around the stator, the other static as it is tethered by two flagella; (c) recovery of GFP-MotB fluorescence after bleaching. (b) and (c) from [17].

In the early 2000s Richard Berry returned to the Physics Department in Oxford after a period with Howard Berg in Harvard. Richard was keen to directly measure how many stators were around a functioning rotor. Richard is exceptionally good at building complex microscopes and total internal reflection (TIRF) microscopes had just been described. In these microscopes laser light hits the bottom of a microscope slide at an angle that causes its reflection, but a region of excitation (an evanescent field) is produced 100 nm into any sample on the slide. This is enough to excite proteins with fluorescent tags in the membrane but not excite naturally fluorescent proteins in the cytoplasm. This was ideal for the motor as we could tether a cell by its flagellar filament using FliC antibodies and pull the motor into the evanescent field – as long as we could tag the stators. This was the very beginning of the use of GFP as a protein tag. Funded by BBSRC, after a lot of painstaking work we made a fully functional genomic MotB-GFP fusion and built a TIRF microscope. We were then able to visualize and count the number of stators. GFP bleaches in laser light and we could use the steps in fluorescence reduction to calculate the original number of stators around a functional *E. coli* motor, at least 11 [17] (Fig. 2a, b).

But then something really unexpected happened. The MotB-GFP proteins should have bleached and stayed bleached, while still driving motor rotation. However, after the initial bleaching, fluorescence started to return (Fig. 2c). This could only happen if there is an exchange of bleached stators for unbleached stators, even though all the previous biochemical and genetic evidence said MotB must be anchored to the peptidoglycan. As a group we were really unsure what was happening and really concerned that something was happening with our fusions or the microscope we had not taken account of. The experiments were repeated again and again, and recalculated again and again, but it became clear that an individual stator only stays associated with a rotor for about 30 s before exchanging with a stator diffusing in the membrane. This was not the result of stators being damaged as we saw fluorescence fall in distant motors presumably as bleached stators engaged and were swapped for unbleached stators. We calculated a pool of around 200 stators are diffusing in the membrane. This was a very controversial set of results at the time, but has since led to revised understanding of the dynamic structure of large protein complexes.

### Where did this lead?

**Fig. 3.**
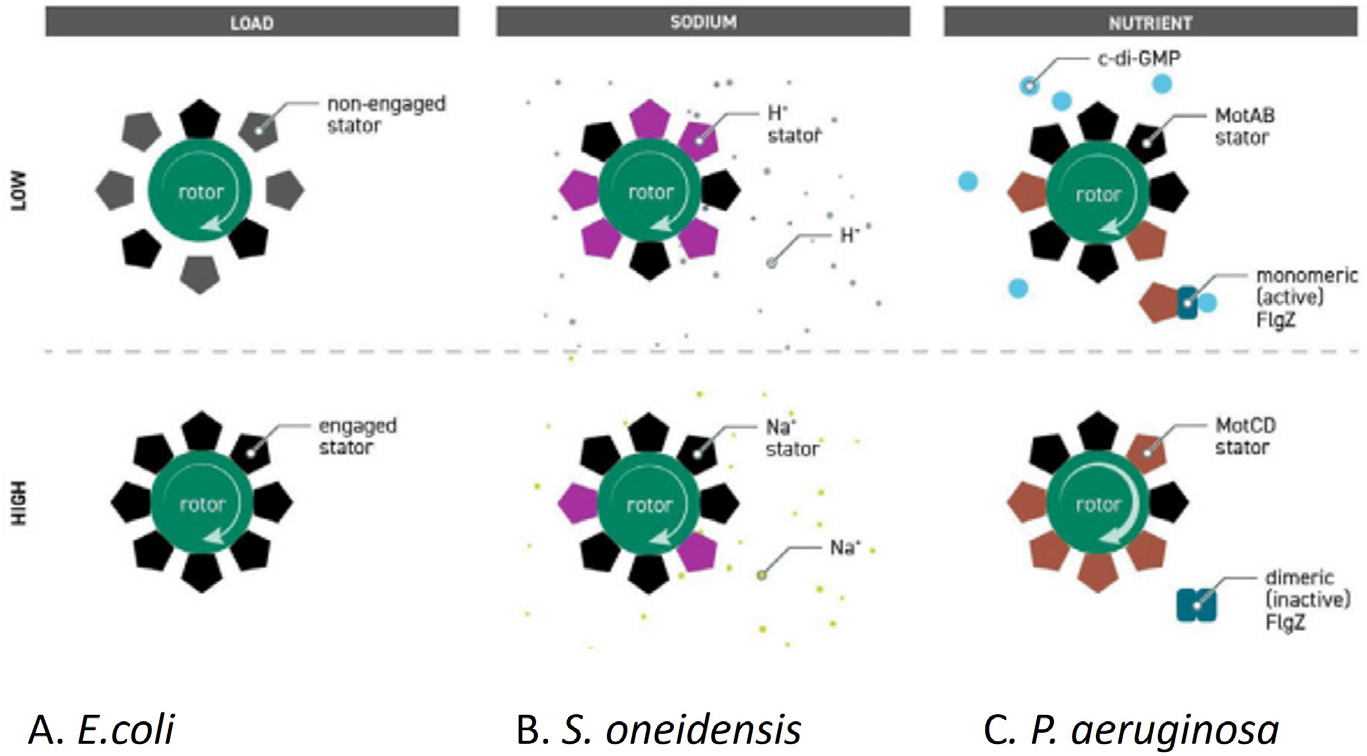
. Environmental control of stator exchange. (a) *E. coli* increasing number of engaged stators with an increase in external viscosity (load); (b) *S. oneidensis* exchanging proton stators for sodium driven stators as concentration of sodium increases; (c) *Ps. aeruginosa* exchanging low-torque stators for high-torque stators on a surface (adapted from [60]).

Following on from those early findings, we went on to show in *E. coli* that (a) if the pmf is dropped to zero and rotation stops, all the stators disengage and diffuse in the membrane, but once the pmf is reinstated the stators return, probably encountering a rotor by chance as they diffuse in the membrane and causing a step-wise increase in rotation as they re-engage [18]; (b) at low external load, i.e. low viscosity, there are very few stators engaged, supporting the idea that each stator can produce near maximum torque, but as the external viscosity increases the number of stators engaged increases, allowing increased torque and continued swimming at full speed. Structural changes in the rotor proteins caused by increased force on the filament probably decreases the stator off rate and thus keeps stators engaged for longer. As the viscosity increases it gets to a point, the stall torque, where the motor can no longer produce enough force to turn the motor and attached filament, but if you wait about 10 mins the motor will start to turn slowly, suggesting an additional stator has engaged (Fig. 3a). This also suggests there are not fixed stator positions around the rotor [19]. (c) To our surprise we also found that it is not just stator proteins that exchange, but FliM and FliN, the chemotaxis switch proteins (see later), associated with the rotor protein FliG also exchange, in this case with cytoplasmic pools of protein [20, 21]. FliG and the membrane spanning associated rotor proteins do not exchange. Recent structural studies suggest the exchange links to a measured decrease in the diameter of the switch ring caused by CheY ~P binding and this altering the interaction between FliG and the stators to drive a switch in rotational direction.

### *Other* species

As some bacterial species appeared to encode more than one set of stator proteins, our findings with the *E. coli* motor structure caused other groups working on different species to investigate the possible roles of multiple stators in those species. Kai Thormann showed that *Shewanella oneidensis* can exchange stators that use sodium ions for proton-driven stators as the sodium concentration falls [22, 23]. Most *Shewanella* species are found in high sodium environments and use a sodium motive force for most energetic processes. *S. oneidensis* was isolated from a fresh-water lake and may have acquired the second, proton-driven, set of stators by horizontal gene transfer and is evolving towards using these stators with the original flagellum, although they are currently less efficient than the sodium stators (Fig. 3b). *Pseudomonas aeruginosa* encodes two different proton-driven stator systems. George O’Toole had shown that one set, MotAB, are used for swimming in liquid and the other set, MotCD, is used in high viscosity media, specifically on surfaces. Although *Ps. aeruginosa* can move on surfaces using pili, it can also use its flagella to crawl through a layer of liquid on a surface, but only if the MotCD stators engage. *Ps. aeruginosa* will swap low-torque generating MotAB stators for high-torque MotCD stators as the viscosity increases, but only if the nutrient levels are high, favouring surface spreading rather than biofilm formation. If however the nutrient levels are low the intracellular concentration of cyclic GMP increases. C-GMP binds to a cytoplasmic protein, FliZ, and it then binds MotCD preventing the MotCD stators binding to the rotor. Without MotCD bound to the rotor the *Ps. aeruginosa* motor cannot rotate, the cells are static. The increase in MotCD-FliZ also appears to induce an increase in the production of extracellular polysaccharide and this plus the inability encourages biofilm formation (Fig. 3c) [24–26].

Using the same techniques, with Andreas Diepold, we found that in addition to flagellar proteins, the evolutionarily related proteins of the type 3 secretion system (T3SS) of *Yersinia* also exchange with cytoplasmic pools and this is linked to secretory signals. Many large protein complexes are almost certainly dynamic and the multiprotein complexes change on activation.

## Explaining earlier data

When we first saw the recovery of fluorescence and were really concerned about whether we were looking at a real phenomenon or not, Richard reminded me of a paper I had written back in 1985 and which he had tried to understand. Part of my research interest has always been the photoheterotrophic bacterium, *Rhodobacter sphaeroides*. It can grow as a heterotroph, but when oxygen levels fall it induces its anoxygenic photosynthetic system. The extent of induction depends on light levels. I am fascinated by its motility and responses to oxygen and light as it is repelled by oxygen when growing photosynthetically and attracted when growing aerobically. We now know this is because both respiratory and photosynthetic electron-transfer systems share some components of the electron-transport chain and a pulse of oxygen to a photosynthetically grown cell causes a transient reduction in electron-transfer rate and this sends a negative signal to the chemotaxis system.

In 1985, using photosynthetically grown cells, I had titrated the proton ionophore, CCCP, to a level where cells swam in the light, but stopped in the dark because the pmf dissipated. When reilluminated, photosynthetic electron transport immediately regenerated the pmf allowing the cells to swim again, but there was a delay. Although the pmf was regenerated instantly, the length of that delay was dependant on the time the cells spent in the dark and this could be up to minutes. I speculated that when the pmf was removed, the structure of the motor might have changed and needed time to reform [27]. This old, then confusing, result fitted with what we were seeing in the TIRF experiments and gave us confidence that what we were seeing was real. The delayed swimming I had seen when the pmf was restored was the result of the stators drifting away from the rotor at zero pmf and needing to re-engage when the pmf was restored.

This plus the results from species encoding one flagellum but more than one type of stator suggest this is a common property of the bacterial flagellum. It also explains why early cryo-EM studies only identified structures thought to be stators in bacterial species with periplasmic flagella, such as spirochetes and leptospira. Their flagella rotate in the periplasmic space to rotate the whole-cell body and are therefore operating under conditions of high viscosity and have their full set of stators permanently engaged.

**The lesson**: Remember old confusing data, the technology might catch up and it can change your career!

## Rhodobacter sphaeroides *and more unexpected results!*

Although I have worked on many bacterial species during my career, I have returned to *R. sphaeroides* time and again. As mentioned above I was initially interested because of its apparent ‘decision making’ connected to growth conditions. It also has a different swimming pattern when compared to *E. coli*, the go-to species of the day. It only has one flagellum, but it is not polar, and the hook, rather than the curved shape of the peritrichous flagellum of *E. coli*, seems thinner than the filament and straight. I assumed it was actively positioned sub-polarly, in a similar way to the active positioning of the polar flagellum of pseudomonads, but detailed analysis of multiple electron micrographs by Chi Aizawa showed the flagella are in fact randomly positioned (personal communication), suggesting there is no precise positioning. *R. sphaeroides* seems to be more like a peritrichous species, but with only one filament. The flagellar regulon is expressed once during the cell cycle and the resulting proteins positioned randomly, possible near the transcription/translation site. A newly divided cell is almost coccoid and has one flagellum. During the ~3 h cell cycle, it elongates and a new filament appears on the daughter cell, never the flagellate cell, just before division. Just before division this doublet of undivided cells spins, as both flagella rotate, until division after which they swim apart.

When watched down a microscope, the swimming pattern of *R. sphaeroides* is a period of smooth swimming followed by a stop, rather than the swim and tumble of peritrichously flagellate species or the swim and transient reverse of polarly flagellate species. A trip to Bob Macnab’s lab at Yale in the 1980s to use a high-intensity dark-field microscope, the only microscope that could visualize working flagella on a swimming bacterium *in vivo* at that time, showed that *R. sphaeroides* does change direction by stopping rotating its flagellum rather than tumbling, which requires a switching of the direction of motor rotation [28]. Stops happen even though the pmf remains high, suggesting either there is a brake or the stators disengage, like a clutch. The frequency of stops changes in chemotactic gradients suggesting some control by intracellular signals (see later). During a stop, the flagellum changed wavelength, relaxing from a functional waveform to a short wavelength, large amplitude filament, which coils close to the cell body. The functional waveform filament reforms from the cell body out as the motor resumes rotation, suggesting that the torque produced by the rotating motor drives the formation of the swimming helix. *R. sphaeroides* was the first species to be identified that shows this swim-stop pattern of motility.

As I said above, a stop with a full pmf would mean the motor had either locked, i.e. applied a brake, or disengaged the stators, similar to putting the foot on the clutch when driving. When we examined the force required to move a stopped cell, tethered by its flagella, using either a laser trap or fluid flow, we found that the force needed to move the cell body is much higher than the force needed to push a non-stopped cell, indicating that during a stop the motor is locked tight, rather than disengaged, i.e. the motor uses a brake not a clutch [29]. We also saw that during a stop there is still a full complement of stator proteins around the rotor, but there is still stator exchange. This suggests strongly that engagement with the rotor and torque development is independent of anchoring and release of the stators from the peptidoglycan, supporting the idea that increased stator engagement with increasing torque changes the stator off-rate.

**Fig. 4.**
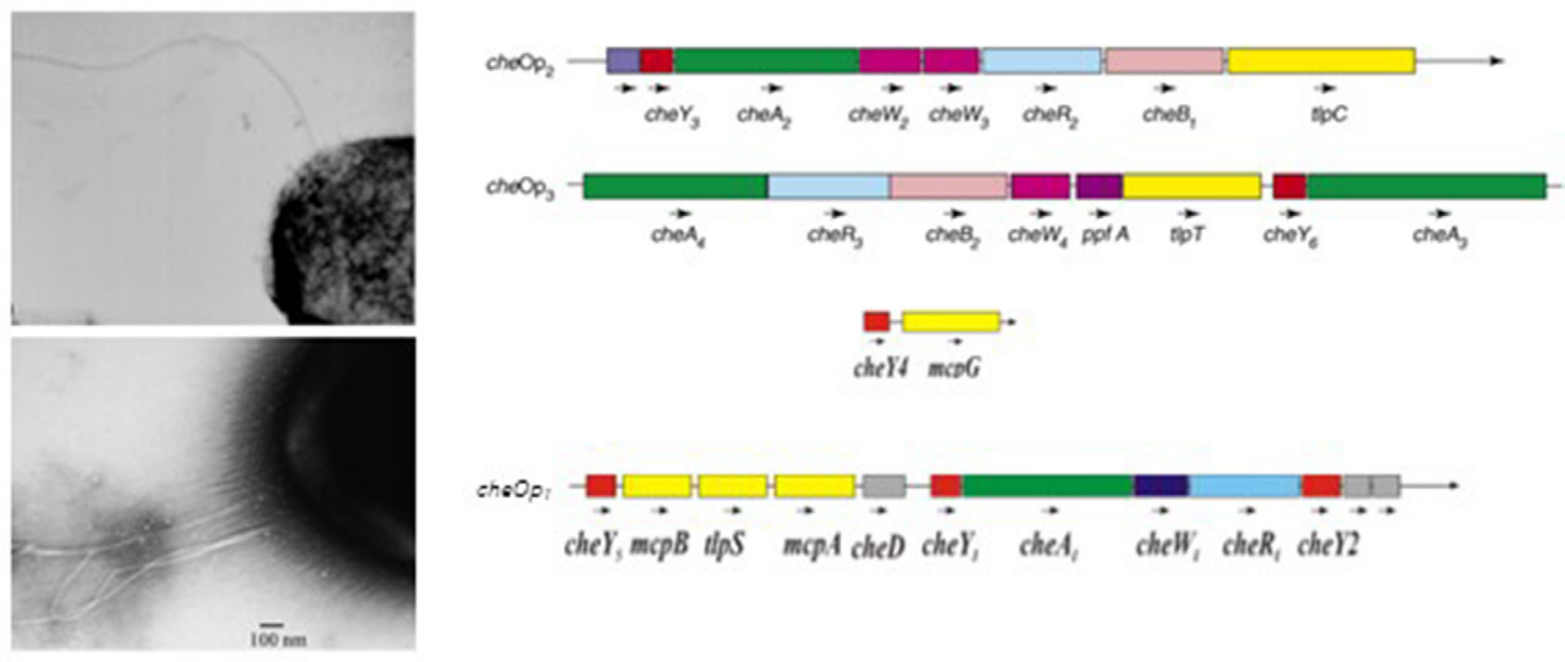
Electron micrographs showing the two flagellar systems of *R. sphaeroides*. Top shows the single randomly positioned, unidirectional flagellum expressed under laboratory conditions while the bottom image shows the polar tuft of flagellar under the control of the Ctr system [35]. Operons show the three systems controlling the flagellar systems. CheOp2 and CheOp3 control the single flagellum while CheOp1, which is not expressed under laboratory conditions, control the polar tuft of flagellar.

Investigations over the years by many groups has shown that while all flagella have a conserved core, there are many variations on a theme. Bacterial species that swim through viscous environments might not only have different stators, they often have larger diameter rotor rings, allowing increased torque. Some species have additional, often periplasmic, rings. These include sodium-driven motors. When George Dreyfus examined the *R. sphaeroides* motor in detail he found that it has an additional periplasmic ring. Whether the extra motor ring links to stopping is unclear [30]. Intriguingly, when the genome of *R. sphaeroides* was sequenced, which at the time took close to 2 years and a great deal of money complicated by it having two chromosomes and a very high GC percentage, regulons for two complete flagellar systems were identified (Fig. 4) [31]. The regulon that looks closest to other alpha proteobacterial flagellar regulons is not expressed under normal laboratory conditions, but the Dreyfus group found that when induced it results in a polar tuft of flagella (Fig. 4) [32]. The single, random, swim-stop flagellum we see normally in the lab appears to be the result of horizontal gene transfer [33–35]. It has not been possible to express the two systems together and the conditions causing switching between the two has not been identified.

I now wonder whether this reflects the artificial environment in which, until recently, we investigated bacterial physiology. Nutrient-rich, shake cultures of single species does not reflect growth in ‘the wild’. *R. sphaeroides*, as a photoheterotroph living in damp soil, the rhizosphere or fresh-water sediments will experience a day/night diurnal rhythm, and changes in, often limiting, nutrient concentrations. More significantly will be the competition with large numbers of neighbouring microbial species, for those limited resources, driving expression of ‘weapons’ from antibiotics and toxins to physical weapons, such as type 6 secretion systems. Plant roots will be secreting nutrients, but there will also be a constant threat of phage. In the highly competitive, and extremely complex natural environment of bacteria it is likely that a very different repertoire of gene expression is required than in the lab. We know *R. sphaeroides* can for biofilms on surfaces, for example, but great efforts are made to prevent biofilm colonies developing in the lab as they complicate extraction processes. I often wonder whether, in the wild, *R. sphaeroides* might swim using that polar tuft of filaments rather than the single stop-start flagellum I spent my career working on!

## Chemotaxis and *R. sphaeroides* again changes the paradigm

When the genome of *R. sphaeroides* was eventually sequenced, it showed not only that it had two completely separate flagellar regulons, but a very complex chemosensory system, probably linked to the acquisition of the alternative flagellum [36]. This helped explain why we had been pretty confused about the behaviour of *R. sphaeroides* for a decade, and showed that we were correct in thinking the chemotaxis pathway must be different from the *E. coli* paradigm that had developed from the late 1960s.

As mentioned earlier, bacteria use temporal not spatial sensing. They compare their environment now with that a few seconds ago, swimming a little longer in a favourable direction and for a shorter time in an unfavourable direction. This requires not only a sensory system but also a memory of the past and an adaptation mechanism enabling the bacterium to sense small changes when continuing to move in a gradient. Most bacterial species tested can sense a very small percentage change in the background concentrations over 5–6 orders of magnitude background concentration.

Back in the 1950s Rod Clayton, working on the photosynthetic bacterium *Rhodospirillum rubrum,* related to *R. sphaeroides,* used a range of elegant competition and growth experiments to suggest that bacterial chemotaxis is linked to metabolic need [37, 38]. This idea however lost ground in the late 1960s when a number of groups started working on chemotaxis in *E. coli* [39]. We now know that the *E. coli* chemotaxis system is one of the simplest in the bacterial world, but, given the lack of molecular tools at the time, studying *E. coli* enabled the field to move forward very rapidly and produced the framework for understand the elegant mechanism by which bacteria navigate their complex environment [40]. However, the downside was that, as with much bacteriology at the time, a strong belief developed that all bacterial species must use the same system as *E. coli*. I freely admit to falling into this trap and spent too much time trying to fit our chemotaxis data into models developed for *E. coli*. It was hard as a young female scientist to contradict the strong beliefs of the numerous, more senior, groups all working on aspects of *E. coli* behaviour. They thought my data were wrong, and I started to believe them.

**Fig. 5.**
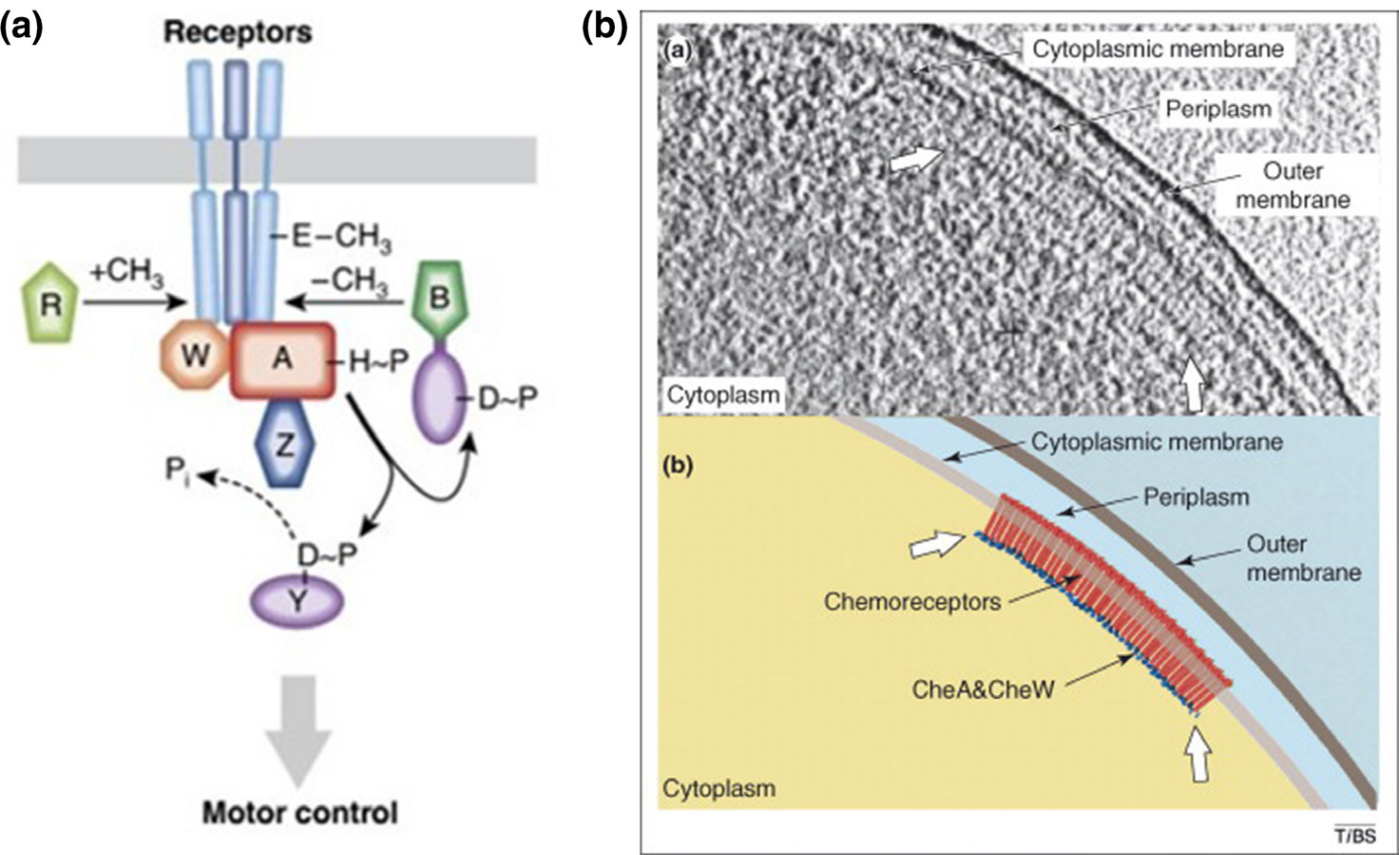
Organization of the *E. coli* chemosensory pathway. On binding or release of attractant a signal propagates through the MCP dimer to inhibit phosphorylation of the HPK, CheA, reducing the level of the signalling response regulator, CheY ~P in the cytoplasm and allowing continued rotation of the motor. Removal of the attractant increases CheA activity and thus the concentration of CheY ~P, which binds the motor causing a change in rotational direction. The rate of CheY ~P dephosphorylation is increased by CheZ while the receptors are reset by a change in activity of the methyl esterase, CheB relative to the transferase, CheR adding and removing methyl groups from conserved glutamate residues on the MCPs [61] . The receptors and associated CheW and CheA proteins form a large lattice near the pole of the membrane as shown in the cryoEM image.

Briefly, *E. coli* has four different, but closely related, types of transmembrane chemoreceptors known as methyl accepting chemotaxis proteins (MCPs) plus an oxygen sensor, Aer. There are thousands of copies of the MCPs forming organized rafts of receptors localized usually close to the poles (Fig. 5). The receptors are all homodimers organized into mixed trimers of dimers and crosslinked at their cytoplasmic tips by a protein CheW and an unusual histidine protein kinase (HPK) CheA. Unlike typical HPKs, the His-binding domain is separated from the kinase domain by a P2 region. Two very different response regulator (RR) proteins compete for binding to the P2 domain, CheY, a single domain RR, and CheB, a methyl esterase involved in adaptation, or resetting, the signalling state of the MCPs. *E. coli*, at least laboratory habituated *E. coli*, responds to a limited number of chemoeffectors, primarily the amino acids serine and aspartate and, through their periplasmic binding protein partners, the sugars ribose and maltose. The amino acids or binding protein bound sugars interact with the periplasmic domain of the MCP and a signal it transmitted across the membrane to CheA bound to the MCP cytoplasmic domain. Loss of a bound chemoeffector results in the CheY bound to CheA being phosphorylated, via ATP, released and diffusing to the flagellar motor. CheY ~P binds the FliM protein of the switch complex of the motor causing a structural change which is transmitted to the rotor protein FliG, changing the charge face interacting with the stator ring (Fig. 5). At a critical tipping point sufficient FliG subunits have changed to cause a switch in direction of rotation. In *E. coli* the rate of CheY ~P dephosphorylation is increased by binding to another protein CheZ. Signalling takes about 100 ms and termination happening within a few seconds. While CheY signals to the motor, the other protein competing for the P2 domain of CheA, CheB, is also phosphorylated and its activity as an esterase increases. MCPs have an extremely long, ~30 nm, cytoplasmic domain made of coil-coils. There are four conserved glutamates which can be methylated by a constitutively active methyl transferase, CheR and demethylated by actived CheB, changing the charge state of the MCP.

This change in methylation state changes local packing, resetting the signalling state of the receptors and acting as a memory of the current environment. Therefore, in response to a decrease in stimulus *E. coli* responds by sending a signal, CheY ~P, from the receptor cluster to the motor, it binds and causes a switch in rotation. CheZ dephosphorylates CheY ~P, terminating the signal and the CheB-CheR enzyme pair reset the receptors allowing subsequent changes to be sensed [41, 42].

Back in the 1990s it was assumed that all bacterial species, including *R. sphaeroides*, would have a similar, single chemosensory pathway with multiple copies of a few transmembrane receptors triggered by a limited number of chemoeffectors. A response would only require binding to MCPs not transport or metabolism, indeed non-transportable and non-metabolizable analogues of attractants cause a chemotactic response. However, when we started looking for compounds that would attract *R. sphaeroides*, or cause a behavioural change when added to swimming cells on a microscope slide, there was no response to identified *E. coli* attractants. Indeed *R. sphaeroides* only responds to metabolites or changes in oxygen or light levels, with the biggest response being to removal not addition. Further investigation indicated that the major attractants are organic acids used in heterotrophic and photoheterotrophic growth and to cause a response the chemoeffector needs to be transported and at least partly metabolized. *R. sphaeroides* appears to be a pessimistic bacterium, responding to a fall in metabolic activity. Our data were met with scepticism, but it did fit with those earlier findings of Clayton working on *R. rubrum* back in the 1950s, but how did this fit with the generally accepted model of *E. coli* chemotaxis?

Things got worse when we started looking for the equivalent genes to those identified in *E*. coli as encoding the chemotaxis pathway. Molecular genetic tools developed rapidly through 1990s. Using the newly developed Southern-blot technology and a painstakingly made cosmid bank of the *R*. *sphaeroides* genome, we went looking to look for genes that might encode a chemosensory pathway, using probes based on a conserved sequence from *E. coli*. We got a strong hit and thought we had the chemotaxis operon. However, when we inserted transposons sequentially into the genes, we got absolutely no change in chemosensory behaviour. Indeed when we finally deleted the whole operon, nothing happened to the swimming or the chemosensory behaviour! In *E. coli* mutating specific steps in the pathway would lead to either smooth swimming or constant tumbling. When we deleted the *R. sphaeroides* operons the cells all continued to swim and stop as though nothing had happened.

These results were greeted with some scepticism and were very difficult to interpret.

As molecular techniques improved all became clear. There was more than one chemotaxis operon, and we had found the only one that is not expressed under normal laboratory conditions! Fascinating looking back, but a nightmare for the postdocs and graduate students involved at the time. Indeed we found another two operons using the cosmid bank. It turns out that *R. sphaeroides* actually has three chemosensory operons (Fig. 4). The one we initially identified and deleted to no avail probably encodes the ancestral pathway and controls the polar tuft of flagella discovered later and described above.

Much to our joy and relief, deleting any of the genes of the other two operons, now called CheOp2 and CheOp3, caused a loss in chemotaxis, showing that both pathways are required for chemotaxis Fig. 4. The two new operons are organized differently from that of *E. coli,* and encode unexpected proteins, multiple copies of CheW and CheY and two putative chemoreceptors without membrane spanning regions. In addition we identified genes that could encode eight classical membrane spanning chemoreceptors. We had hoped that one of the chemoreceptors might have a binding site for a redox sensor or chromophore, expected of oxygen or light sensors, but none have.

One of the initial reasons for investigating *R*. *sphaeroides* behaviour had been the responses to light and oxygen. Very early on I had seen cells attracted to oxygen when grown heterotrophically but repelled by oxygen if grown photoheterotrophically. Cells also stop and change direction when swimming over a light-dark boundary but not a dark-light, resulting in accumulation in light, and if a spectrum is shone onto a microscope slide they accumulate in actinic wavelengths, including far red, suggesting photosynthesis might be actively involved. Having identified the additional chemosensory operons, we found that CheOp2 is required for responses to both oxygen and light. By artificially manipulating the electron-transport chain and controlling changes in the pmf we were able to show that *R. sphaeroides* does not have specific oxygen or light receptors, as identified in other species, but responds to a transient drop in the rate of electron transport, sensed through the terminal cytochrome Cbb_3_ and the chemosensory system [43]. A pulse of oxygen to a photoheterotrophic cell reduces electron transport through the shared respiratory and photosynthetic components of the chain, resulting in a drop in membrane potential and direction changing, preventing *R. sphaeroides* moving into oxygenated environments when growing photosynthetically. Which protein senses the redox change in the electron-transport chain to signal through CheOp2 has not been identified.

**Fig. 6.**
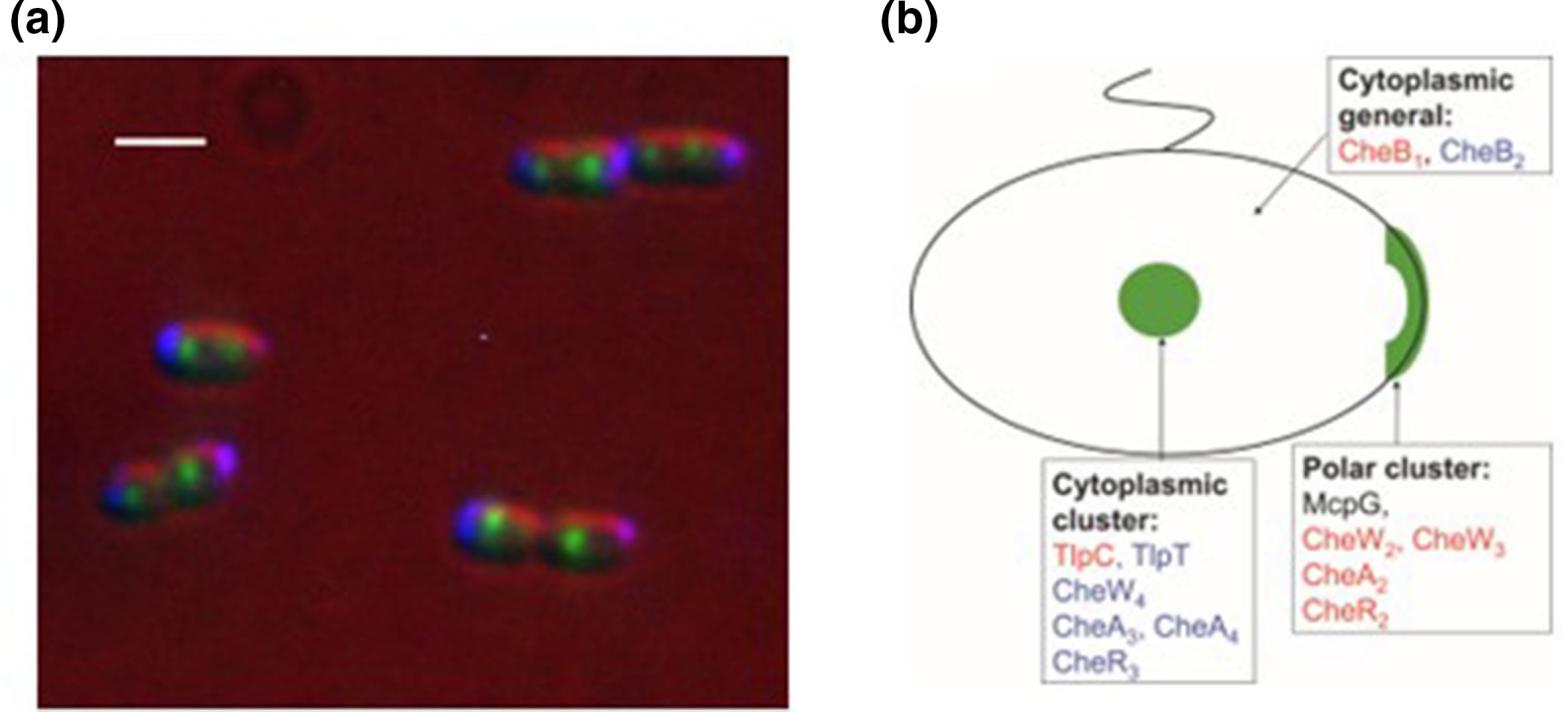
Localization of the proteins of CheOp2 and CheOp3 in *R. sphaeroides*. (a) Membrane localized and cytoplasmic chemosensory clusters via fluorescently labelled receptors. Newly divided cells have a single cluster and as cells grow polar clusters grow and cytoplasmic cluster divides and each daughter inherits cluster. (b) Different proteins targeted to the differently positioned pathways. Blue proteins are encoded by CheOp3 and red by CheOp2 [48].

Given this complexity of pathways and that immunoelectron microscopy showed that the *E. coli* chemoreceptors are mainly in large clusters at the poles, we decided to see where the *R*. s*phaeroides* receptors localized and whether the proteins from the two functioning operons were mixed. Immunogold EM showed that *R. sphaeroides* indeed had clusters of receptors at the poles, but in addition there was always either one or two clusters in the cytoplasm [44]. Making functional genomic GFP fusions to each of the proteins encoded in the two expressed pathways revealed that all the proteins encoded by CheOp2, except TlpC, localize to the poles and all the proteins encoded by CheOp3, including TlpC and TlpT are in one or two tight cytoplasmic clusters, the number depending on the growth phase of the cell (Fig. 6) [45]. Initially we expected that as each operon apparently encoded a complete chemosensory pathway that apparently homologous proteins encoded by the two expressed pathways would be complementary, but to our surprise we found that both pathways are essential for chemotaxis and apparently homologous proteins cannot complement for the lack of each other, even when swapped between operons to ensure correct expression levels and localization [46, 47]. The cytoplasmic chemoreceptors, in particular TlpT, also turned out to be essential for chemotaxis, although deletion of any of the membrane spanning MCPs only caused a minor reduction in response. The essential nature of TlpT shows that transmembrane signalling is not a necessary part of the behavioural response.

By this time we had enough strong data that we were being believed, but at conferences I would still be relegated to the ‘funny bugs’ section on the last day!

How are the different proteins of the two pathways organised, in particular the cytoplasmic cluster? How do they come together into one large multiprotein structure, does the second cluster form *de novo* as the cell grows or does the single cluster split, segregating so that each cell has a cluster on division and, critically, how do MCP-like receptors signal without the supposedly critical transmembrane domain triggering the change in kinase activity? CheOp3 encodes a chemoreceptor without a transmembrane domain, TlpT, and two unusual CheAs, CheA3 and CheA4. It took us far longer to get to grips with these two CheAs than it should have done, and again I admit it is down to a blinkered belief that all CheAs would look like that of *E. coli*.

Before we had a complete genome sequence we used our cosmid bank to search for and then clone and sequence putative chemotaxis genes. We found a gene that we thought could encode a CheA, *cheA3*, and we sequenced it repeatedly, convinced we were making errors as the gene was far too long and although it encoded a classical His domain and an MCP-binding domain there was no kinase domain, instead there was an extremely long central domain with no homologue in any database. In the same region was a gene that could encode another apparent CheA, *cheA4*, again with an MCP-binding domain, but in this case a kinase-binding domain but no His-binding domain [48]. I am ashamed to say that I did not have the courage to believe our data as it ran so counter to all the published data. Only when we had full, reliable genome sequences was it clear that those original sequences were correct, *R. sphaeroides* CheOp3 does encode two very unusual CheA homologues. Both have MCP-binding domains, but one has the kinase domain and the other the His domain, suggesting they function together, possibly activated by different chemoreceptors. Large-scale bioinformatic studies by the Zhulin lab of thousands of genome sequences has shown that these really are two very unusual CheAs and supporting the idea of recent acquisition and evolutionary adaptation of this pathway to control the single flagellum.

Detailed genetic and biochemical analysis of the purified proteins showed that both CheAs are essential for chemotaxis and CheA4 does phosphorylate CheA3. While the CheA, CheA2, associated with the membrane cluster can phosphorylate all CheYs and CheBs, including those of the unexpressed CheOp1, showing it to be a ‘classical’ CheA. CheA3 however only phosphorylates one*,* CheY6, encoded in the same operon, and CheB2. The extremely long internal domain of CheA3 increases the dephosphorylation rate of CheY6 ~P, but no other CheY, taking on the role of CheZ in *E. coli*. Extensive studies showed that, despite CheY3 and CheY4 being phosphorylated by CheA2, and being required for chemotaxis, neither can stop the motor. Only CheY6 ~P can stop the motor.

**Fig. 7.**
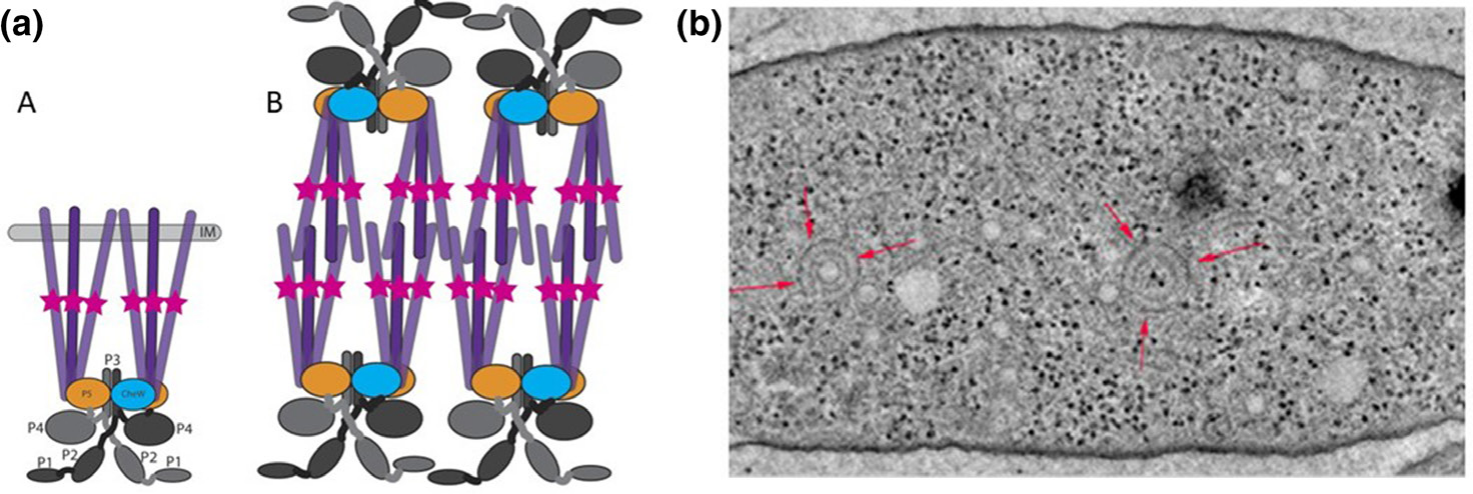
Cryo-EM showing two cytoplasmic chemosensory cluster, marked by arrows. Drawing shows position of chemosensory proteins. (a) The organization of the membrane-associated proteins; purple-receptors; grey-CheA, with CheY and CheB associated; stars adaptation sites. (b) Arragment in *R. sphaeroides* cytoplasmic cluster showing overlapping receptor tips where the membrane sits in the membrane cluster [62].

While working out the complex pattern of which protein could interact with which other protein to control behaviour, we continued to look at the position and organization of the two complexes, in particular the two MCP-like proteins without transmembrane receptors. Cryo- electron microscopy went some way to addressing this as it revealed a double layer of receptors with the tips of proteins overlapping to form a sandwich-like array of receptors producing a curved complex in the cytoplasm with the tips of the receptors overlapping and signalling proteins on the outside of the sandwich (Fig. 7).

When followed through the cell cycle, the chemosensory cluster, which appears fixed in one region of the cell, grows in size and at a certain point appears to divide and one portion moves to the daughter cell so that each cell has a cytoplasmic cluster on division.

The CheOp3 sequence includes an unexpected gene, not apparently associated with chemotaxis that we call *PpfA*. This protein strong sequence similarity to the plasmid partitioning protein ParA [49]. We ignored the gene for a long time because deletion only caused a reduction in chemotaxis, not loss and we assumed it be a polar effect on downstream expression. It was not until we looked at the position of fluorescently labelled chemotaxis proteins that PpfA really is involved in chemotaxis. Deletion of PpfA results in one very large cytoplasmic chemotaxis cluster that moves randomly around the cell, suggesting that it might be involved in anchoring the cluster. On division one daughter inherits this large cluster, but within 30 min or so a new cluster starts to be visible in the other daughter cell, showing there is no effect on expression of CheOp3 genes. Deletion of the receptor TlpT however resulted in the CheOp3 proteins diffusing in the cytoplasm and not forming a cluster. Expressing TlpT from an inducible plasmid caused the clusters to reform, evenly spaced at about one cell intervals, even in cephalexin induced filaments [50]. PpfA is a ParA-like protein that associates with the nucleoid surface, even that of *E. coli*. Unlike ParA and plasmid portioning, PpfA is randomly spread over the chromosome surface and not dynamic. TlpT binds to PpfA, and TlpT nucleates the formation of the chemosensory cluster. The equal spacing along the chromosome is the result of a simple, passive reaction diffusion system, hence the even spacing seen in filaments. We hypothesize that as the chromosome replicates the DNA polymerase moves through the chromosome, splitting the CheOp3 cluster and then one portion of the cluster moves with each chromosome, thus ensuring each daughter cell inherits a chemotaxis cluster. Extensive studies and modelling has found no evidence of an active mechanism, suggesting a purely passive use of the chromosome as the vehicle for piggy-backing to ensure cluster segregation on division. Like many others, I discovered the publishing world is not very interested in passive processes.

**Fig. 8.**
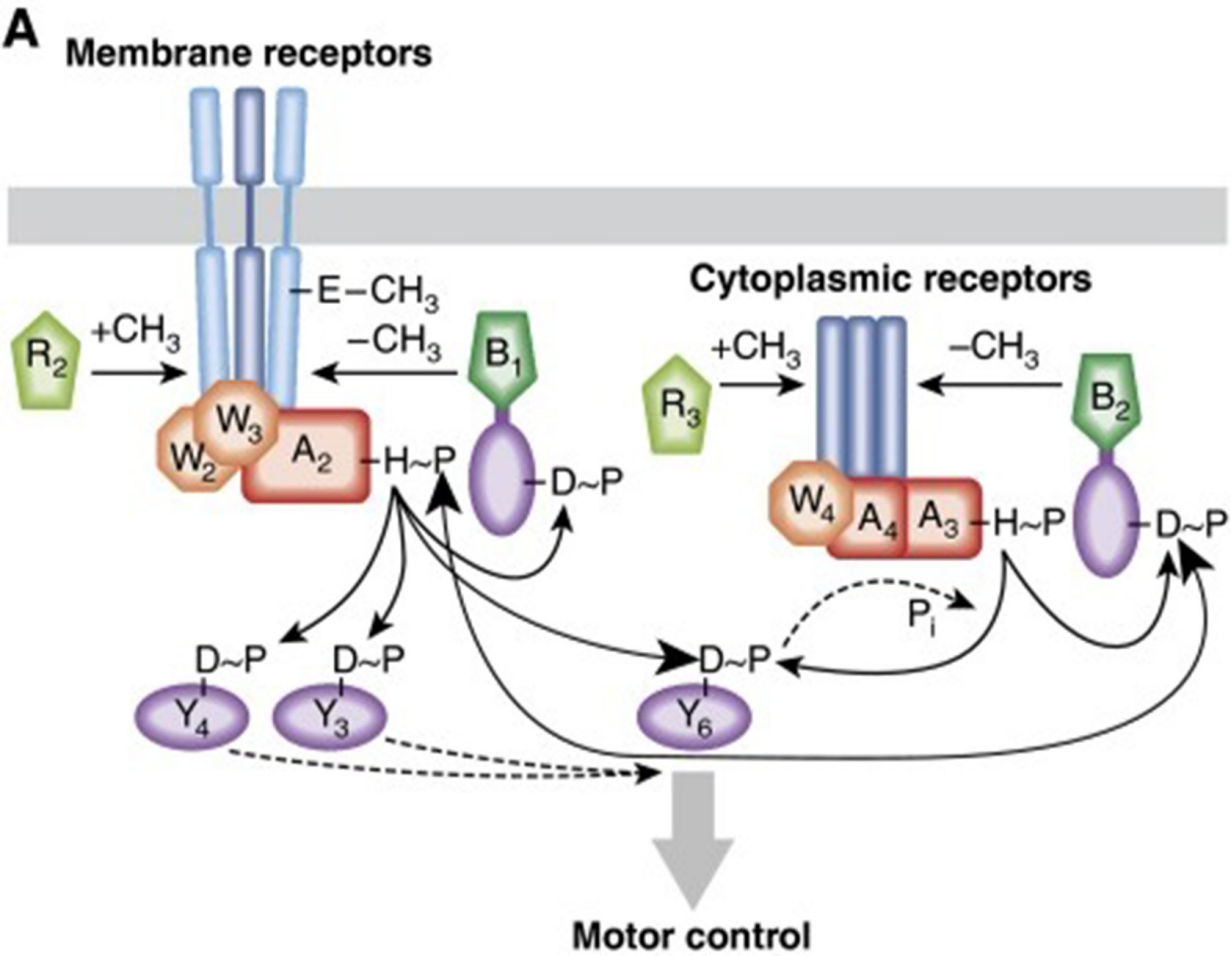
Simplified model of chemosensory signalling in *R. sphaeroides*. Cytoplasmic chemosensors sense metabolic state and tune the responsiveness of the motor through concentration of motor binding of CheY6 ~P. CheY6 ~P is the only Chey to stop the motor, but CheY3/4~P can compete CheY6 ~P off the motor to allow smooth swimming. The motor stop-start frequency depends on the relative concentrations of the CheY ~Ps responding to internal and external stimuli to allow a balanced response [61].

The key cytoplasmic, chromosome-localizing receptor TlpT also controls the activity of CheA3 in response to cytoplasmic rather than extracellular signals. CheA3 is specifically phosphorylated by another unusual CheA, CheA4, which has a kinase domain but no histidine domain. Its kinase activity probably controlled by a second cytoplasmic receptor TlpC. CheA3 is phosphorylated by CheA4 in response to a chemoeffector. CheA3 then both phosphorylates, and dephosphorylates, the key motor-binding CheY, CheY6 [51–53]. It seems probable that the metabolic state of the cell acts on TlpT to change the phosphatase activity of CheA3 and thus the level of CheY6 ~P in the cell. Models suggest that the balance of kinase to phosphatase activity controls the level of CheY6 ~P in the cell and this tunes the stopping frequency of the cell relative to metabolic state (Fig. 8) [54].

*R. sphaeroides* does however respond to transient changes in the external environment sensed through the membrane receptors. CheY3 ~P and CheY4 ~P released from CheA2 bound to the MCP receptors can bind the motor and can compete CheY6 ~P off the motor, resulting in smooth swimming but a drop in metabolic state results in CheY6 ~P increasing and competing them off and the motor stopping. As with transmembrane MCP receptors, adaptation to a new steady state is important. There is little evidence that TlpC shows modification, but TlpT does adapt and resets CheA3 phosphotransfer and dephosphorylation rates. Adaptation of TlpT is essential for chemotaxis and depends on CheR3 and CheB2 methylating and demethylating unusual glutamates on TlpT [55].

*R. sphaeroides* therefore appears to respond to a nutrient gradient only if there is a change in metabolic state, usually a reduction. Unlike *E. coli* which has a limited capacity to decide whether to follow a gradient of aspartate, ever when saturated by serine, *R. sphaeroides* responds only when its metabolic state changes, keeping or moving it to an optimum environment for growth. One real frustration is that we have not identified the TlpT effector. It seems possible this links to the global redox state of the cell, but that remains for others to identify.

## An aside: intracellular diffusion signalling and segregating chromosomes

*R. sphaeroides* has been a model for studies of photosynthetic biophysics over the years, but it has many more fascinating physiological traits. When thinking about chemotaxis we wondered whether response times were the same under aerobic and photoheterotrophic conditions and were surprised to discover that while the responses to percentage changes were the same, the threshold for responding and response times differ. We wondered whether the fact that photoheterotrophic grown cells become filled with 50 nm photosynthetic vesicles could have an effect on diffusion rates of cytoplasmic proteins. Using FRET sensors and single-cell tracking we found that these vesicles do indeed impede the cytoplasmic diffusion of CheY6 and other proteins [56]. This suggests that we need to take into account any morphological changes in bacterial cells when investigating behaviour.

Do these vesicles have an effect on other cellular processes such as chromosome segregation? This is particularly interesting as *R. sphaeroides* has two chromosomes, the large (~3 Mb) C1 chromosome with *oriC* site at one pole and *ter* site at the other, and the small C2 (~1 Mb) with the *oriC* and *ter* sites at midcell. Tracking the movement of the segregation and cell-division proteins revealed a complex choreography of proteins through the 3 h cell cycle, rather different from that of other alpha proteobacteria. Unlike other alpha proteobacteria OriC1 and ParB1 localize to the old pole, and MipZ and FtsZ at the new pole. ParB1 moving to the new pole following chromosome duplication releases FtsZ, which forms a ring at midcell while MipZ monomers oscillate between poles, with nucleotide-bound monomers and dimers localizing to midcell. Unexpectedly MipZ dimers form a ring close to the FtsZ ring and constrict with the FtsZ ring suggesting that MipZ dimers are regulating FtsZ activity and septation [57]. What path does the OriC/ParB complex, with the duplicating chromosome, take though the vesicle packed cytoplasm? What other cellular activities might be affected? This is for others to find.

## Summary

The linked subjects of bacterial swimming and controlling that swimming through chemotaxis has taken my research in two interconnected directions, one leading to studies of macromolecular structures and protein dynamics in general and the other to the complexity of signal transduction and cellular physiology in *R. sphaeroides*. Both strands required grasping new techniques as they became available and interdisciplinary collaboration. I could not have made the breakthroughs or developed the new insights without the biophysicists, mathematical and computational modellers and image analysists who worked alongside my exceptionally dedicated team of molecular biologists and biochemists. What is clear is that each new result and insight is just a step along the road. It is the best interpretation at the time and hopefully in the future others will be intrigued by the questions still to be answered and have a career as fascinating and rewarding as mine. Very early in my career Pat Clarke, only the second female microbiologist to be made an FRS, advised me to not work in a ‘fashionable’ area as the easy questions had been answered and, as a young woman, it would be difficult to be accepted. She advised me rather to identify a new, developing area that really interested me, and make it my own. If I was really interested, I could make others interested and a career would follow. Does this advice apply today with the intense competition for funding? I think it does, particularly when applying for early career fellowships. Experience on several different panels has shown me that panels are looking for scientists who are not only passionate about their chosen area, but want to build on their experience and have the insight to see possible new and exciting directions. In these days of mega-labs and big expensive kit starting your own lab is daunting and the time to make your mark short, so you need to become visible through the crowd of established researchers also after those limited funds. Having an innovative angle on your area, collaborating with researchers in different fields with new technologies or having absorbed a range of approaches that have added new dimensions and broadened your thinking beyond the rest of the field will make you stand out. My career was built on interdisciplinary collaboration. It provided not only a new take on an area, it gave me knowledgeable, rigorous collaborators who helped me overcome those who doubted our research.

At the beginning of my career I started working on *Rhodopseudomonas sphaeroides*, named in 1944 by van Neil, but in 1984, after I have written my first few papers, it was reclassified as *Rhodobacter sphaeroides*. In 2020 it was again reclassified, this time as *Cereibacter sphaeroides*. I quite like the idea that the species name has retired with me!
